# Preventable Hospitalizations and Emergency Department Visits for Angina, United States, 1995–2010

**DOI:** 10.5888/pcd10.120322

**Published:** 2013-07-25

**Authors:** Julie C. Will, Amy L. Valderrama, Paula W. Yoon

**Affiliations:** Author Affiliations: Amy L. Valderrama, Paula W. Yoon, Centers for Disease Control and Prevention, Atlanta, Georgia.

## Abstract

**Introduction:**

Preventable hospitalizations for angina have been decreasing since the late 1980s — most likely because of changes in guidance, physician coding practices, and reimbursement. We asked whether this national decline has continued and whether preventable emergency department visits for angina show a similar decline.

**Methods:**

We used National Hospital Discharge Survey data from 1995 through 2010 and National Hospital Ambulatory Medical Care Survey data from 1995 through 2009 to study preventable hospitalizations and emergency department visits, respectively. We calculated both crude and standardized rates for these visits according to technical specifications published by the Agency for Healthcare Research and Quality, which uses population estimates from the US Census Bureau as the denominator for the rates.

**Results:**

Crude hospitalization rates for angina declined from 1995–1998 to 2007–2010 for men and women in all 3 age groups (18–44, 45–64, and ≥65) and age- and sex-standardized rates declined in a linear fashion (*P* = .02). Crude rates for preventable emergency department visits for angina declined for men and women aged 65 or older from 1995–1998 to 2007–2009. Age- and sex-standardized rates for these visits showed a linear decline (*P* = .05).

**Conclusion:**

We extend previous research by showing that preventable hospitalization rates for angina have continued to decline beyond the time studied previously. We also show that emergency department visits for the same condition have also declined during the past 15 years. Although these declines are probably due to changes in diagnostic practices in the hospitals and emergency departments, more studies are needed to fully understand the reasons behind this phenomenon.

## MEDSCAPE CME

Medscape, LLC is pleased to provide online continuing medical education (CME) for this journal article, allowing clinicians the opportunity to earn CME credit.

This activity has been planned and implemented in accordance with the Essential Areas and policies of the Accreditation Council for Continuing Medical Education through the joint sponsorship of Medscape, LLC and Preventing Chronic Disease. Medscape, LLC is accredited by the ACCME to provide continuing medical education for physicians.

Medscape, LLC designates this Journal-based CME activity for a maximum of 1 **AMA PRA Category 1 Credit(s)™**. Physicians should claim only the credit commensurate with the extent of their participation in the activity.

All other clinicians completing this activity will be issued a certificate of participation. To participate in this journal CME activity: (1) review the learning objectives and author disclosures; (2) study the education content; (3) take the post-test with a 70% minimum passing score and complete the evaluation at www.medscape.org/journal/pcd (4) view/print certificate.

**Release date: July 24, 2013; Expiration date: July 24, 2014**

### Learning Objectives

Upon completion of this activity, participants will be able to:

- Evaluate trends in emergency department visits for chest pain
- Analyze characteristics of emergency department visits for angina in the current study
- Analyze characteristics of hospitalizations for angina in the current study
- Distinguish patterns in preventable emergency department visits and hospitalizations for angina over time

**EDITORS**

Caran Wilbanks, Editor, *Preventing Chronic Disease*. Disclosure: Caran Wilbanks has disclosed the following relevant financial relationship: Partner is employed by McKesson Corporation.

**CME AUTHOR**

Charles P. Vega, MD, Associate Professor and Residency Director, Department of Family Medicine, University of California, Irvine. Disclosure: Charles P. Vega, MD, has disclosed no relevant financial relationships.

**AUTHORS AND CREDENTIALS**

Disclosures: Julie C. Will, PhD, MPH, owns stock, stock options, or bonds from Johnson & Johnson and Pfizer. Amy L. Valderrama, PhD,and Paula W. Yoon, ScD, MPH have disclosed no relevant financial relationships.

Affiliation: Julie C. Will, Amy L. Valderrama, and Paula W. Yoon, Centers for Disease Control and Prevention, Atlanta, Georgia.

## Introduction

Expert committees identified conditions for which hospitalization could be avoided if patients had early access to good quality outpatient health care (1,2), and the experts labeled these conditions as ambulatory care-sensitive conditions or preventable hospitalizations. Preventable hospitalizations for angina are believed to capture the failure of the outpatient health care system to prevent and control cardiovascular disease risk factors (3). For patients with coronary and other atherosclerotic diseases, clinical guidelines suggest that aggressive, comprehensive risk factor management is likely to improve these patients’ lives by reducing the number of procedural interventions they require — possibly resulting in reduced hospitalizations (4).

Secular declines in preventable hospitalizations for angina have been reported for people aged 65 years or older (5). Other researchers also reported declines for some angina hospitalizations (6,7). At least 1 researcher (7) suggested that this decrease may partially be due to increased use of emergency departments (EDs) or outpatient clinics to manage acute chest pain. Others have suggested, on the basis of observing increasing rates of hospitalization for coronary atherosclerosis and increased use of coronary angiography during 1992 through 1999, that the decline is not related to better access to care or improvements in preventive care. Instead, they believe that it reflects trends in more aggressive diagnosis of coronary atherosclerosis that have led to different discharge diagnoses (5).

The purpose of this study was to confirm previous findings of a decline in preventable hospitalizations for angina as defined by Agency for Healthcare Research and Quality’s (AHRQ’s) Prevention Quality Indicator (PQI) number 13 (8), by using a different national database, a more recent time period, and both younger and older adult patients. Furthermore, this study examined whether rates of potentially preventable ED visits for angina have increased over time, lending support to the idea that cases are declining in the hospital setting because they are being handled more often in the ED.

## Methods

### Data source and definitions

We obtained hospitalization data (1995–2010) from the National Hospital Discharge Survey (NHDS). We obtained ED visit data (1995–2009) from the National Hospital Ambulatory Medical Care Survey (NHAMCS). Both surveys were conducted by the National Center for Health Statistics (NCHS) (9,10). All research activities related to the surveys were reviewed and approved by the NCHS Research Ethics Review Board in accordance with 45 CFR 46. NHDS and NHAMCS are stratified, probability-designed surveys; the NHDS has 3 stages of sampling and the NHAMCS has 4 stages. Although the sampling frames and methods are different for the 2 surveys, at the first and second stages of sampling the surveys select noninstitutional hospitals in geographic areas of the United States, exclusive of federal, military, and Veterans Administration hospitals. Only short-stay hospitals (those with an average length of stay <30 days for all patients) and general hospitals (medical or surgical) are included in the surveys. Hospitals must also have at least 6 beds available for inpatient use. At the final stage, surveys select a systematic random sample of patient visits from either the participating hospital (NHDS) or the ED (NHAMCS). During 2008 through 2010, NCHS used half samples for the NHDS because of limited financial resources.

Response rates for the NHDS hospitals are generally at least 90%; however, in 2008, 2009, and 2010 the rates dropped to about 86%. In NHAMCS, about 90% of hospitals respond to the survey. From the hospitals with EDs, approximately 90% of EDs agree to provide survey information.

We calculated NHDS hospitalization and NHAMCS ED rates according to the technical specifications published by AHRQ for PQI number 13 (8). The numerator consists of all nonmaternal discharges of people aged 18 years or older with an *International Classification of Diseases, Ninth Revision, Clinical Modification* (ICD-9 CM) principal diagnosis code for angina: intermediate coronary syndrome, including unstable angina (411.1), acute coronary occlusion without myocardial infarction (411.81), acute ischemic heart disease (411.89), angina decubitus (413.0), prinzmetal angina (413.1), or angina pectoris (413.9). From both databases, we excluded maternal discharges using a method based on ICD-9 codes alone (11). Other exclusions required by the AHRQ specifications include transfers from another institution (the NHDS database is missing this information before 2001) and discharges with cardiac procedure codes in any field. In the ED analysis, we did not exclude these because transfers into an ED have a different meaning from transfers into a hospital, and cardiac procedures of this type are not generally done in the ED. By excluding cardiac procedures, the number of hospitalizations is reduced by approximately 10% and more serious and complex cases are eliminated. For example, persons who require procedures such as a cardiac pacemaker implant, cardiac valve repair, or coronary bypass are not included in the numerator. The denominators for the rates are from US Census population estimates (12) published by NCHS as part of the documentation package for each year’s survey database (9,10).

For NHDS, after the survey staff selected discharge records for study, demographic and medical data were abstracted by US Bureau of the Census staff (acting as agents for NCHS) or by hospital staff primarily from the records’ face sheets (the first page or cover page containing information such as health and medical requirements listed in an easy-to-use format) and discharge summaries. Editing and quality checks were made by NCHS; then the records were made available for analysis via a computerized database. We used the confidential database at the NCHS’s Research Data Center (which includes variables not available in the NHDS public-use database) for this analysis, which allowed us to use sampling design variables to calculate standard errors. For NHAMCS, hospital staff used medical records to complete the Patient Record Forms (brief, one-page forms that record the required survey information) for the ED visits. We used the public-use database because it included sampling design variables along with demographic and medical information.

In describing the characteristics of patients with preventable visits, we used 3 categories for race: white, black, or other (13). In calculating rates, we did not stratify by race because of sample size concerns. We used 4 US Census regions: Northeast, Midwest, South, and West (12). For insurance, we used only the principal expected source of payment to derive 4 categories; Medicare, Medicaid, private insurance, and other. “Other” included types such as other government insurance, self-pay, hospitalization without a charge, and worker’s compensation. Source of payment was considered missing and excluded from the calculation of percentages if the medical record abstract form or face sheets had “not stated” checked.

### Statistical analysis

We estimated the total weighted number of preventable hospitalizations or ED visits for angina each year for persons aged 18 years or older. Because angina as a primary reason for a visit is rare, we combined 4 years of data to obtain 4 time periods: 1995–1998, 1999–2002, 2003–2006, and 2007 through the most recent year of data available (2010 for NHDS and 2009 for NHAMCS). We summed the census population estimates during these same time periods and used the results to calculate rates per 100,000 population.

We stratified the population by sex and age (18–44, 45–64, and ≥65 years for NHDS; and 18–64 and ≥65 years for NHAMCS). We used only 2 age categories for NHAMCS because using 3 resulted in large standard errors for point estimates. We also produced age- and sex- standardized rates using the 2000 US Census population as the standard population (12).

We used Proc Crosstab in SUDAAN 10.0 (Research Triangle Institute, Research Triangle Park, North Carolina) to calculate 95% confidence intervals (CIs) around the estimates. We did not calculate CIs around the denominators because they were derived from a census of the population. We tested differences between subgroups using *z* tests with α = .05 as a measure of significance; however, this testing was only done for estimates that were considered reliable according to NCHS standards (ie, estimates based on 30 or more visits and those where the relative standard error is 30% or less). We used the weighted least squares regression test for linear trends for each study subgroup and for the age- and sex-specific rates (14). To examine the assumption of linearity, we examined residuals plotted against the independent variable to verify that the residuals were randomly distributed.

## Results

### Hospitalizations

From 1995 through 2010, there were 13,962 records for which angina without procedure was the first-listed diagnosis among persons aged 18 years or older in the NHDS sampled hospitals. This translates to a weighted estimate of 1,926,000 of these hospitalizations for US adults during the 16 years, an average of 120,000 each year.

In 1995–1998, most hospitalizations (57.2%) occurred among persons aged 65 years or older (Table 1); however, this changed by 2007–2010, when there were nonsignificant differences in the percentages between those aged 45 to 64 years (48.1%; 95% CI, 41.9%–54.4%) and those aged 65 years or older (39.7%; 95% CI, 33.7%–46.1%). The distribution of hospitalizations did not vary significantly by sex. From 1995 through 2002, hospitalizations for angina without procedure occurred less frequently in the western US Census region than in other regions. The largest payer for these hospitalizations was Medicare, ranging from 49% to 55% depending on the time period. Private insurance was the second largest payer, ranging from 31% to 35%. In 2003–2006, Medicaid paid for approximately 8% of these hospitalizations.

**Table 1:** Preventable Hospitalizations for Angina, Weighted Number of Visits and Percentage of Visits Within 4 Time Periods, by Selected Demographic Characteristics, National Hospital Discharge Survey, 1995–2010

Characteristics	1995–1998		1999–2002		2003–2006		2007–2010	
	No.	% (95% CI)	No.	% (95% CI)	No.	% (95% CI)	No.	% (95% CI)
**Age, y**								
18–44	67,747 (55,186–80,308)	7.9 (6.9–9.1)	42,409 (33,738–51,080)	7.9 (6.6–9.4)	30,482 (23,001–37,963)	8.7 (7.0–10.7)	22,237 (13,741–30,733)	12.1 (8.8–16.6)
45–64	298,789 (270,421–327,157	34.9 (32.3–37.6)	215,405 (181,615–249,195)	40.2 (36.9–43.6)	158,838 (137,316–180,360)	45.2 (41.0–49.4)	88,118 (69,479–106,757)	48.1 (41.9–54.4)
≥65	489,188 (425,915–552,461	57.2 (54.5–59.8)	278000 (230,606–325,394)	51.9 (48.4–55.4)	162,489 (134,597–190,381)	46.2– (41.8–50.6)	72,765 (53,931–91,599)	39.7 (33.7–46.1)
Total	855,724 (767,796–943,652)	100.0	535,814 (458,679–612,949)	100.0	351,809 (308,441–395,177)	100.0	183,120 (148,541–217,699)	100.0
**Sex**								
Male	411,336 (364,490–458,182)	48.1 (46.0–50.2)	258,773 (220,173–297,373)	48.3 (44.7–51.9)	173,275 (150,620–195,930)	49.3 (46.2–52.3)	91,450 (72,280–110,620)	49.9 (44.4–55.5)
Female	444,388 (396,160–492,616)	51.9 (49.8–54.0)	277,041 (229,455–324,627)	51.7 (48.1–55.3)	178,534 (152,794–204,274)	50.8 (47.7–53.8)	91,670 (70,685–112,655)	50.1 (44.5–55.6)
Total	855,724 (767,796–943,652)	100.0	535,814 (458,679–612,949)	100.0	351,809 (308,441–395,177)	100.0	183,120 (148,541–217,699)	100.0
**Race**								
White	577,591 (501,848–653,334)	83.7 (80.8–86.1)	358,001 (304,634–411,368)	84.3 (81.4–86.7)	225,827 (181,214–270,440)	80.5 (76.2–84.1)	115,794 (88,962–142,626)	75.3 (68.3–81.2)
Black	81,027 (66,692–95,362)	11.7 (9.7–14.1)	48,856 (39,423–58,289)	11.5 (9.5–13.9)	42,125 (34,507–49,743)	15.0 (12.1–18.5)	30,990 (19,295–42,685)	20.2 (14.3–27.6)
Other	31,856 (23,348–40,364)	4.6 (3.5–6.0)	18,086 (11,393–24,779)	4.3 (2.9–6.1)	12,755 (7,383–18,127)	4.5 (3.0–6.9)	7,036 (2,855–11,217)	4.6 (2.6–8.0)
Total^a^	690,474 (611,853–769,095)	100.0	424,943 (368,152–481,734)	100.0	280,707 (234,245–327,169)	100.0	153,820 (121,992–185,648)	100.0
**Region**								
Northeast	265,283 (208,654–321,912)	31.0 (26.1–36.4)	162,512 (100,692–224,332)	30.3 (22.6–39.4)	94,527 (63,605–125,449)	26.9 (20.6–34.2)	50,430 (29,266–71,594)	27.5 (19.2–37.8)
Midwest	209,803 (157,011–262,595)	24.5 (19.8–29.9)	116,232 (83,619–148,845)	21.7 (16.7–27.7)	76,125 (56,555–95,695)	21.6 (17.1–27.0)	31,174 (20,145–42,203)	17.0 (11.9–23.7)
South	266,470 (228,821–304,119)	31.1 (27.1–35.5)	192,298 (163,317–221,279)	35.9 (30.2–42.0)	126,461 (107,715–145,207)	36.0 (30.9–41.4)	67,618 (46,498–88,738)	36.9 (28.4–46.4)
West	114,168 (96,307–132,029)	13.3 (11.3–15.7)	64,772 (50,078–79,466)	12.1 (9.4–15.4)	54,696 (40,910–68,482)	15.6 (12.1–19.7)	33,898 (20,539–47,257)	18.5 (12.7–26.2)
Total	855,724 (767,796–943,652)	100.0	535,814 (458,679–612,949)	100.0	351,809 (308,441–395,177)	100.0	183,120 (148,541–217,699)	100.0
**Insurance status**								
Medicare	463,416 (402,132–524,700)	55.1 (52.2–58.0)	272,705 (226,098–319,312)	51.2 (47.8–54.6)	171,287 (142,697–199,877)	49.4 (44.8–53.9)	80,199 (63,191–97,207)	44.8 (40.3–49.3)
Medicaid	56,257 (46,254–66,260)	6.7 (5.7–7.9)	35,840 (27,670–44,010)	6.7 (5.6–8.0)	27,186 (21,007–33,365)	7.8 (6.4–9.6)	21,662 (13,923–29,401)	12.1 (8.8–16.4)
Private insurance	262,713 (234,482–290,944)	31.3 (28.9–33.7)	185,239 (154,902–215,576)	34.8 (31.3–38.4)	119,110 (99,001–139,219)	34.3 (29.9–39.0)	57,541 (43,423–71,659)	32.1 (27.1–37.6)
Other	58,161 (47,263–69,059)	6.9 (5.9–8.2)	38,858 (29,779–47,937)	7.3 (6.0–8.9)	29,356 (22,542–36,170)	8.5 (6.8–10.5)	19,711 (13,341–26,081)	11.0 (8.2–14.6)
Total^b^	840,547 (754,288–926,806)	100.0	532,642 (455,712–609,572)	100.0	346,939 (304,984–388,894)	100.0	179,113 (146,721–211,505)	100.0

a Totals here are less than totals for the other characteristics because of missing values: 165,250 (19.3%) in 1995–1998; 110,871 (20.7%) in 1999–2002; 71,102 (20.2%) in 2003–2006; and 29,300 (16.0%) in 2007–2010. Missing values were not included in the calculation of the percentages.

b Totals here are less than totals for the other characteristics because of missing values: 15,177 (1.8%) in 1995–1998; 3,172 (0.6%) in 1999–2002; 4,870 (1.4%) in 2003–2006; and 4,007 (2.2%) in 2007–2010. Missing values were not included in the calculation of the percentages.

For both women and men, the rates for preventable angina hospitalizations increased significantly by age within each time period except for the most recent time period (Table 2). Rates dropped significantly from the earlier years (1995–1998) to the later years (2007–2010) for both sexes in every age group (65%–87.7% for women, 70%–85% for men). The highest rates for both sexes occurred from 1995 through 1998 among those aged 65 years or older (women, 287.4/100,000; men, 293.1/100,000).

**Table 2:** Age- and Sex-Stratified Preventable Hospitalizations for Angina per 100,000 Population Among Those Aged 18 Years or Older, by Time Period, National Hospital Discharge Survey, United States, 1995–2010

Age, y/Sex	1995–1998, no. (95% CI)	1999–2002, no. (95% CI)	2003–2006, no. (95% CI)	2007–2010, no. (95% CI)
**18–44**				
Men	15.6^a^ (11.5–19.7)	10.0^a^ (7.6–12.4)	6.8 (4.8–8.9)	4.6^b^ (1.9–7.3)
Women	9.1 (6.5–11.7)	4.7 (2.7–6.8)	4.6 (2.7–6.5)	3.2^b^ (1.4–5.0)
**45–64**				
Men	127.9^a^ (111.3–144.5)	74.6 (61.0–88.2)	49.6 (40.5–58.8)	25.1^b^ (18.5–31.8)
Women	98.9 (84.6–113.1)	62.1 (51.3–72.9)	37.2 (28.3–46.1)	23.1^b^ (16.0–30.1)
**≥65**				
Men	293.1 (247.1–339.0)	156.5 (128.1–184.9)	89.4 (65.7–113.2)	42.9^b^ (28.6–57.1)
Women	287.4 (245.9–329.0)	157.1 (124.1–190.2)	89.7 (68.8–110.7)	35.4^b^ (22.5–48.3)

Abbreviation: CI, confidence interval.

a The male–female difference in this age group and time period is significant at *P* < .05.

b Rates dropped significantly (*P* < .05) from the first time period to the last time period.

### Emergency department visits

During 1995–2009, there were 1,796 preventable ED visits for angina among persons aged 18 years or older in the EDs sampled by NHAMCS. This translates to a weighted number of 6,854,508 of these visits for US adults during 15 years, an average of 457,000 each year.

In 1995–1998, most visits (58.9%) were by persons aged 65 years or older (Table 3); however, this changed by 2007–2009, when the differences in the percentages between those aged 18–64 years (54.6%; 95% CI, 47.1%–61.9%) and those aged 65 years or older (45.4%; 95% CI, 38.1%–52.9%) became nonsignificant. The distribution of ED visits did not vary significantly by sex. As would be expected because of the racial composition of the United States, whites had the most visits. The largest payer for these ED visits was Medicare, which paid for about 50% of them during each study period. Private insurance was the second largest payer covering from 31% to 35% of the visits. In 2003–2006 (the most recent period with a reliable estimate for Medicaid), Medicaid paid for approximately 9% of these ED visits.

**Table 3:** Preventable Emergency Department Visits for Angina, Weighted Number of Visits and Percentage of Visits Within 4 Time Periods, by Selected Demographic Characteristics, National Hospital Ambulatory Medical Care Survey, 1995–2009

Characteristics	1995–1998		1999–2002		2003–2006		2007–2009	
	No.	% (95% CI)	No.	% (95% CI)	No.	% (95% CI)	No.	% (95% CI)
**Age, y**								
18–64	921,264 (732,979–1,109,549)	41.1 (35.7–46.7)	1,054,389 (792,710–1,316,068)	50.4 (44.3–56.6)	827,861 (629,751–1,025,971)	56.6 (50.8–62.3)	579,303 (419,944–738,662)	54.6 (47.1–61.9)
≥65	1,319,642 (1,087,176–1,552,108)	58.9 (53.3–64.3)	1,035,827 (790,004–1,281,650)	49.6 (43.4–55.7)	634,108 (479,831–788,385)	43.4 (37.7–49.2)	482,114 (345,446–618,782)	45.4 (38.1–52.9)
Total	2,240,906 (1,901,893–2,579,919)	100.0	2,090,216 (1,653,952–2,526,480)	100.0	1,461,969 (1,153,769–1,770,169)	100.0	1,061,417 (811,535–1,311,299)	100.0
**Sex**								
Male	1,192,931 (968,809–1,417,053)	53.2 (48.2– 58.2)	1,053,092 (810,450–1,295,734)	50.4 (45.2– 55.6)	724,786 (553,894–895,678)	49.6 (43.9–55.3)	546,920 (399,843–693,997)	51.5 (43.7–59.3)
Female	1,047,975 (865,643–1,230,307)	46.8 (41.8–51.8)	1,037,124 (792,403–1,281,845)	49.6 (44.4–54.8)	737,183 (557,121–917,245)	50.4 (44.7–56.1)	514,497 (361,312–667,682)	48.5 (40.7–56.3)
Total	2,240,906 (1,901,893–2,579,919)	100.0	2,090,216 (1,653,952–2,526,480)	100.0	1,461,969 (1,153,769–1,770,169)	100.0	1,061,417 (811,535–1,311,299)	100.0
**Race**								
White	1,914,937 (1,597,954–2,231,920)	85.5 (81.1–88.9)	1,860,013 (1,443,863–2,276,163)	89.0 (85.0–92.0)	1,230,359 (952,283–1,508,435)	84.2 (79.1–88.2)	867,173 (643,864–1,090,482)	81.7 (75.2–86.8)
Black	269,949 (182,901–356,997)	12.1 (8.8–16.3)	204,632 (134,457–274,807)	9.8 (6.9–13.8)	192,501 (123,837–261,165)	13.2 (9.4–18.2)	153,806 (90,985–216,627)	14.5 (9.8–20.9)
Other	56,020^a^ (24,991–87,049)	2.5 (1.4–4.3)	25,571^a^ (2,046–49,096)	1.2^a^ (0.5–2.9)	39,109^a^ (10,492–67,726)	2.7^a^ (1.3–5.3)	40,438^a^ (10,050–70,826)	3.8^a^ (1.8–7.7)
Total	2,240,906 (1,901,893–2,579,919)	100.0	2,090,216 (1,653,952–2,526,480)	100.0	1,461,969 (1,153,769–1,770,169)	100.0	1,061,417 (811,535–1,311,299)	100.0
**Region**								
Northeast	656,593 (457,969–855,217)	29.3 (22.6–37.0)	424,030 (251,184–596,876)	20.3 (13.7–29.0)	433,192 (275,712–590,672)	29.6 (21.2–39.7)	253,649 (144,204–363,094)	23.9 (15.8–34.4)
Midwest	559,429 (379,101–739,757)	25.0 (18.8–32.4)	541,165 (320,041–762,289)	25.9 (17.8–36.0)	337,055 (189,747–484,363)	23.1 (15.4–33.1)	247,900 (122,417–373,383)	23.4 (14.7–35.1)
South	618,463 (459,837–777,089)	27.6 (21.7–34.3)	750,554 (446,805–1,054,303)	35.9 (26.0–47.2)	408,993 (230,330–587,656)	28.0 (19.1–39.0)	337,587 (184,227–490,947)	31.8 (21.5–44.2)
West	406,421 (272,991–539,851)	18.1 (13.3–24.3)	374,467 (235,572–513,362)	17.9 (12.3–25.4)	282,729 (153,994–411,464)	19.3 (12.5–28.6)	222,281 (116,461–328,101)	20.9 (13.3–31.4)
Total	2,240,906 (1,901,893–2,579,919)	100.0	2,090,216 (1,653,952–2,526,480)	100.0	1,461,969 (1,153,769–1,770,169)	100.0	1,061,417 (811,535–1,311,299)	100.0
**Insurance status**								
Medicare	1,172,809 (956,094–1,389,524)	52.6 (47.3–57.8)	1,025,258 (768,155–1,282,361)	49.3 (43.6–54.9)	648,214 (485,171–811,257)	44.7 (38.8–50.8)	496,279 (365,927–626,631)	48.6 (41.4–55.9)
Medicaid	127,981^a^ (69,436–186,526)	5.7 (3.7–8.8)	155,414 (88,748–222,080)	7.5 (5.0–10.9)	131,911 (83,255–180,567)	9.1 (6.5–12.6)	84,442^a^ (14,069–154,815)	8.3^a^ (3.8–17.0)
Private insurance	691,293 (528,540–854,046)	31.0 (26.1–36.3)	733,390 (544,932–921,848)	35.2 (29.4–41.5)	443,700 (309,184–578,216)	30.6 (25.2–36.5)	357,366 (248,430–466,302)	35.0 (29.5–40.9)
Other	238,795 (164,560–313,030)	10.7 (7.9–14.4)	167,803 (97,138–238,468)	8.1 (5.6–11.5)	226,953 (148,968–304,938)	15.6 (11.5–21.0)	83,145^a^ (41,088–125,202)	8.1^a^ (4.9–13.2)
Total^b^	2,230,878 (1,893,710–2,568,046)	100.0	2,081,865 (1,646,211–2,517,519)	100.0	1,450,778 (1,145,748–1,755,808)	100.0	1,021,232 (777,016–1,265,448)	100.0

a This estimate is unreliable according to the National Center for Health Statistics’ standard that estimates should be based on either 30 or more records or a relative standard error of 30% or less.

b Totals here are less than totals for the other characteristics because of missing values: 10,028 in 1995–1998; 8,351 in 1999–2002; 11,191 in 2003–2006; and 40,185 in 2007–2009. Missing values are not included in the calculation of the percentages.

For women and men, the rates for preventable ED visits for angina were significantly higher for those aged 65 years or older compared with those who were younger (Table 4). Among those aged 65 or older, rates dropped significantly from the earlier years (1995–1998) to the later years (2007–2009) for both sexes in every age group (50% for women, 67% for men). The highest rates for both sexes occurred in 1995–1998 among those aged 65 years or older (women, 933.2/100,000; men, 1,170.5/100,000).

**Table 4:** Age- and Sex-Stratified Preventable Emergency Department Visits for Angina per 100,000 Population Among Those Aged 18 Years or Older, by Time Period, National Hospital Ambulatory Medical Care Survey, United States, 1995–2009

Age,y/Sex	1995–1998	1999–2002	2003–2006	2007–2009
**18–64**				
Men	177.3 (131.5–223.04)	177.6 (131.5–223.7)	126.0 (89.7–162.2)	129.4 (92.5–166.4)
Women	107.7 (79.6–135.8)	129.6 (86.0–173.1)	102.3 (75.3–129.2)	77.0 (43.3–110.8)
**≥65**				
Men	1,170.5 (874.8–1,466.3)	806.1 (582.7–1,029.6)	461.7 (327.4–595.9)	391.2^a^ (242.1–540.3)
Women	933.2 (746.3–1,119.9)	753.2 (546.8–959.7)	448.2 (317.4–578.9)	464.1^a^ (308.0–620.2)

a Rates dropped significantly (*P* < .05) from the first period to the last period.

### Linear trends

A test of linear trend shows that age- and sex-standardized hospitalization rates for angina (Figure) declined in a significant linear fashion (β = −21/100,000, *P* = .02). The actual rate declined from 89.1 per 100,000 to 15.8 per 100,000 — an 82% decline. Standardized rates for ED visits also declined in a significant linear fashion (β = −48/100,000, *P* = .05). The actual rates declined from 291.5 per 100,000 to 158.6 per 100,000 — a 46% decline.

**Figure:**
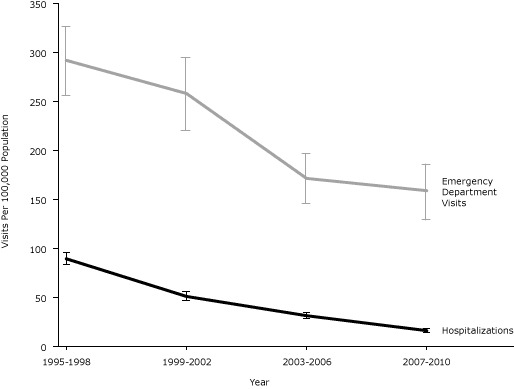
Rates of age- and sex-standardized preventable hospitalization and emergency department visits for angina in the United States across 4 time periods. Sources: National Hospital Discharge Survey, 1995–2010 (9) and National Hospital Ambulatory Medical Care Survey, 1995–2009 (10). Data for emergency department visits in the last study time period (2007–2010) are for 2007 through 2009 only. **Table:** 

Year	Visits Per 100,00 Population	
	Hospital	Emergency Department Visits
**1995–1998**	89.1	291.6
**1999–2002**	50.69	257.8
**2003–2006**	31.03	171.1
**2007–2010**	15.76	158.6

## Discussion

We found that both preventable hospitalizations and ED visits for angina declined from the mid-1990s through 2007–2010; however, the rate of decline has been less for ED visits than for preventable hospitalizations. Consequently, the decline in preventable hospitalizations for angina in the inpatient setting is unlikely to be due to increased management of this condition in EDs.

Hypotheses for this rapid decline include 1) a decreasing rate of angina (although little evidence is available, US and British studies reported either flat or increasing rates during the 1990s) (15–17); 2) declining heart disease risk factors that are well documented (18); 3) changing provider practices such as increased testing with troponin (possibly leading to reclassifying angina as a myocardial infarction based on the test results), increased use of revascularization (use of certain procedures disqualify angina as a PQI), or changes in diagnostic practices (such as cardiac catheterization) in the in-patient setting (19); 4) increasing use of EDs or outpatient clinics to manage acute chest pain; and 5) changing hospital ICD-9 CM coding (5,7). Saver and colleagues (5) believe that this decline reflects trends in more aggressive diagnosis of coronary atherosclerosis, which has led to a decline in angina as a primary diagnosis. Research by Bertoni and colleagues (7) shows that it is not just a matter of having moved angina from its position as a primary diagnosis to a nonprimary diagnosis — angina recorded as any diagnosis also declined during 1988–2001.

We explored changes in the rate of preventable ED visits for angina to address the hypothesis that management of angina has moved from the inpatient setting to the ED setting. In fact, ED use has increased over the last 10 or more years with inpatient beds decreasing over this same time period (20). Although noninjury ED visits for chest pain have increased on average about 55,000 each year in the United States during the past decade (which is not a significant increase) (21), our study shows that the rate of preventable ED visits for angina has decreased over time. This leads us to agree with others (5) that more aggressive diagnostic workups have likely led to different discharge diagnoses. This is further supported by 1) the increasing prevalence of observation units in EDs, with more than one-third of hospitals having such a unit in 2007–2008 (22); 2) the fact that chest pain is the leading complaint that results in observation during an ED visit (22); and 3) the increasing use of advanced medical imaging for noninjury chest pain (21). Diagnostic workups of patients with chest pain will likely result in primary discharge diagnoses more specific than angina, such as coronary atherosclerosis or acute coronary syndrome. Accredited chest pain centers have been developed throughout the United States, which has led to better adherence to clinical guidelines for cardiac care (23). The growth of observation units and the use of aggressive diagnostics are often driven by factors such as overcrowding in EDs, failure to be reimbursed after admitting certain patients to the hospital (eg, for very short-term stays), and the urgent need to rule out serious conditions such as a heart attack or stroke (22).

Many patients who in the past would have received diagnoses of angina may now be more likely to receive diagnoses of coronary atherosclerotic disease (5) — a condition that is still considered manageable, if not preventable, in outpatient and community settings. Highly recommended evidence-based management strategies in the outpatient setting include using aspirin therapy to lower low-density lipoprotein cholesterol to targets based on initial risk and using renin-angiotensin-aldosterone system blockers and β-blockers for selected at-risk patients (4). Other recommended strategies include smoking cessation, blood pressure control, physical activity, improved nutrition, weight management, and diabetes management (4). Aggressive risk factor management and therapeutic lifestyle changes for patients with existing coronary and other atherosclerotic vascular disease improve survival, reduce recurrent events, and improve quality of life (24–27).

Our study contributes newly to the literature in the following ways: 1) hospitalization data are from a representative sample of US hospitals; 2) data on preventable ED visits are also examined, leading us to conclude that these visits have declined over time; 3) CIs around rates show the degree of certainty or uncertainty; and 4) our results add almost another decade of data to those of previous studies.

Our study also has limitations. First, it is important to continue to examine our proposed hypotheses and those of other researchers (5–7) using data sets that have more complete information on both admission and discharge diagnoses. These types of data would allow for a greater understanding of what percentage of admission diagnoses of angina are converted to another diagnosis at discharge. The data are currently insufficient to do this because NHDS started collecting admitting diagnoses only in 2007, and NHAMCS does not collect any admitting diagnosis (it collects patient-reported reason for visit). Second, as required by AHRQ’s definition of a PQI, we were unable to exclude angina hospitalizations that occurred because of transfers from another facility for 1995 through 2000 because transfer data were not collected during those years. However, based on those years for which data were available, we believe that lack of transfer data creates only a small error, given that transfers accounted for 0.97%–2.08% of angina hospitalizations. Finally, we did not exclude transfers or cardiac procedures from preventable ED visits, which is routinely done for preventable hospitalizations. So, we are unable to claim that our rates across settings are truly comparable. With the increase in observation units in EDs, the line between hospitalization and ED visits will continue to blur. To get a complete picture of preventable hospitalizations, preventable ED visits also must be studied; to achieve this, more research is needed on the validity of the definition used in this study.

Regardless of the reasons for the decline in angina hospitalizations and ED visits, primary and secondary prevention strategies should continue to be a hallmark of care for patients with coronary artery disease. Using prevention strategies, however, does not obviate the need for further research. To move beyond speculation, studies are needed on factors that influence doctors to change primary admissions diagnoses of angina to primary discharge diagnoses other than angina. Second, validity studies or experts’ opinions are important to provide guidance on the best way to define a preventable ED visit. With these advances, we could more easily determine whether using the metrics of preventable hospitalizations and ED visits for angina will provide valuable information on the quality of outpatient medical care and the ability of the public health system to support primary prevention efforts.

## Figures and Tables

**Table:** 

**1. The activity supported the learning objectives.**				
**Strongly Disagree**				**Strongly Agree**
1	2	3	4	5
**2. The material was organized clearly for learning to occur.**				
**Strongly Disagree**				**Strongly Agree**
1	2	3	4	5
**3. The content learned from this activity will impact my practice.**				
**Strongly Disagree**				**Strongly Agree**
1	2	3	4	5
**4. The activity was presented objectively and free of commercial bias.**				
**Strongly Disagree**				**Strongly Agree**
1	2	3	4	5

## Post-Test Information

To obtain credit, you should first read the journal article. After reading the article, you should be able to answer the following, related, multiple-choice questions. To complete the questions (with a minimum 70% passing score) and earn continuing medical education (CME) credit, please go to http://www.medscape.org/journal/pcd. Credit cannot be obtained for tests completed on paper, although you may use the worksheet below to keep a record of your answers. You must be a registered user on Medscape.org. If you are not registered on Medscape.org, please click on the "Register" link on the right hand side of the website to register. Only one answer is correct for each question. Once you successfully answer all post-test questions you will be able to view and/or print your certificate. For questions regarding the content of this activity, contact the accredited provider, CME@medscape.net. For technical assistance, contact CME@webmd.net. American Medical Association's Physician's Recognition Award (AMA PRA) credits are accepted in the US as evidence of participation in CME activities. For further information on this award, please refer to http://www.ama-assn.org/ama/pub/about-ama/awards/ama-physicians-recognition-award.page. The AMA has determined that physicians not licensed in the US who participate in this CME activity are eligible for **AMA PRA Category 1 Credits™**. Through agreements that the AMA has made with agencies in some countries, AMA PRA credit may be acceptable as evidence of participation in CME activities. If you are not licensed in the US, please complete the questions online, print the AMA PRA CME credit certificate and present it to your national medical association for review.

## Post-Test Questions

### Article Title: Preventable Hospitalizations and Emergency Department Visits for Angina, 1995-2010

**CME Questions**

1. You are seeing a 57-year-old man in the emergency department (ED) for a 2-hour history of left chest pain. Which of the following statements regarding ED visits for chest pain and their management is most accurate?
  - Rates of total non-injury ED visits for chest pain have declined in the past decades
  - The rate of discharge for the diagnosis of chest pain vs more specific pathologic conditions has increased dramatically
  - Observation units are present in less than 5% of EDs
  - Chest pain is the leading cause of admission to the ED observation unit
2. Based on the data in the current study by Dr. Will and colleagues, what should you consider regarding trends in ED visits for angina?
  - By 2007-2009, most visits were among patients at age 65 years or older
  - Preventable ED visits were more common among older vs younger adults
  - Men had more ED visits compared with women
  - Medicaid paid for the majority of ED visits
3. You consider admitting the patient to the hospital for a cardiac workup. What should you consider regarding data on hospitalizations for angina in the current study?
  - Older adults were more likely to be hospitalized throughout the study period
  - Hospitalizations were most common in the western United States
  - Most hospitalizations were covered by Medicare
  - The rate of preventable hospitalizations decreased with time among men, but not women
4. What were the trends in preventable ED visits and hospitalizations for angina in the current study?
  - Reductions in the rates of both preventable ED visits and hospitalizations over time
  - Higher rates of preventable ED visits but lower rates of preventable hospitalizations over time
  - Lower rates of preventable ED visits over time, but no change in the rate of preventable hospitalizations
  - Higher rates of both preventable ED visits and preventable hospitalizations over time

**Evaluation**

## References

[1] Billings J , Zeitel L , Lukomnik J , Carey TS , Blank AE , Newman L . Impact of socioeconomic status on hospital use in New York City. Health Aff (Millwood) 1993;12(1):162–73. 10.1377/hlthaff.12.1.162 8509018

[2] Weissman JS , Gatsonis C , Epstein AM . Rates of avoidable hospitalizations by insurance status in Massachusetts and Maryland. JAMA 1992;268(17):2388–94. 10.1001/jama.1992.03490170060026 1404795

[3] Guide to prevention quality indicators: hospital admission for ambulatory care sensitive conditions. AHRQ Publication No. 02-R0203. Rockville (MD): Agency for Health Care Research and Quality; 2007. http://www.qualityindicators.ahrq.gov/Downloads/Modules/PQI/V31/pqi_guide_v31.pdf. Accessed January 31, 2013.

[4] Smith SC Jr , Allen J , Blair SN , Bonow RO , Brass LM , Fonarow GC , AHA/ACC guidelines for secondary prevention for patients with coronary and other atherosclerotic vascular disease: 2006 update: endorsed by the National Heart, Lung, and Blood Institute. Circulation 2006;113(19):2363–72. Erratum in Circulation 2006;113(22):e847. 10.1161/CIRCULATIONAHA.106.174516 16702489

[5] Saver BG , Dobie SA , Green PK , Wang CY , Baldwin LM . No pain, but no gain? The disappearance of angina hospitalizations, 1992–1999. Med Care 2009;47(10):1106–10. 10.1097/MLR.0b013e31819e1f53 19820615 PMC2761607

[6] Davis SK , Liu Y , Gibbons GH . Disparities in trends of hospitalization for potentially preventable chronic conditions among African Americans during the 1990s: implications and benchmarks. Am J Public Health 2003;93(3):447–55. Erratum in Am J Public Health 2003;93(5):703. 10.2105/AJPH.93.3.447 12604494 PMC1447762

[7] Bertoni AG , Bonds DE , Thom T , Chen GJ , Goff DC Jr . Acute coronary syndrome national statistics: challenges in definitions. Am Heart J 2005;149(6):1055–61. 10.1016/j.ahj.2004.10.040 15976788

[8] Prevention Quality Indicators technical specifications, PQI#13 Angina without procedure admission rate. Version 4.1. Rockville (MD): Agency for Health Care Research and Quality; 2009. http://www.qualityindicators.ahrq.gov. Accessed November 19, 2012.

[9] National Hospital Discharge Survey questionnaires, datasets, and related documentation. US Department of Health and Human Services, Centers for Disease Control and Prevention; 2013. http://www.cdc.gov/nchs/nhds/nhds_questionnaires.htm. Accessed June 5, 2013.

[10] Ambulatory Health Care Data questionnaires, datasets, and related documents. US Department of Health and Human Services, Centers for Disease Control and Prevention; 2013. http://www.cdc.gov/nchs/ahcd/ahcd_questionnaires.htm. Accessed June 5, 2013.

[11] Kuklina EV , Whiteman MK , Hillis SD , Jamieson DJ , Meikle SF , Posner SF , An enhanced method for identifying obstetric deliveries: implications for estimating maternal morbidity. Matern Child Health J 2008;12(4):469–77. 10.1007/s10995-007-0256-6 17690963

[12] Census 2000 brief series. Washington (DC): US Census Bureau; 2000. http://www.census.gov/population/www/cen2000/briefs.html. Accessed November 19, 2012.

[13] Kozak LJ . Underreporting of race in the National Hospital Discharge Survey. Adv Data 1995;(265):1–12. 10154340

[14] Gillum BS , Graves EJ , Jean L . Trends in hospital utilization: United States, 1988–92. National Center for Health Statistics. Vital Health Stat 13 1996;(124):1–71. 8764687

[15] Ford ES , Giles WH . Changes in prevalence of nonfatal coronary heart disease in the United States from 1971–1994. Ethn Dis 2003;13(1):85–93. 12723017

[16] McCormick A , Fleming D , Charlton J ; Royal College of General Practitioners. Morbidity statistics from general practice, fourth national study, 1991–1992. London (GB): Office of Population Censuses and Surveys, Department of Health and Social Security; 1995.

[17] Lampe FC , Morris RW , Walker M , Shaper AG , Whincup PH . Trends in rates of different forms of diagnosed coronary heart disease, 1978 to 2000: prospective, population based study of British men. BMJ 2005;330(7499):1046. 10.1136/bmj.330.7499.1046 15879388 PMC557220

[18] Capewell S , Ford ES , Croft JB , Critchley JA , Greenlund KJ , Labarthe DR . Cardiovascular risk factor trends and potential for reducing coronary heart disease mortality in the United States of America. Bull World Health Organ 2010;88(2):120–30. 10.2471/BLT.08.057885 20428369 PMC2814476

[19] Hall MJ , DeFrances CJ , Williams SN , Golosinskiy A , Schwartzman A . National Hospital Discharge Survey: 2007 summary. National Health Statistics Reports 29; 2010. http://www.cdc.gov/nchs/data/nhsr/nhsr029.pdf. Accessed September 17, 2012. 21086860

[20] Kellermann AL . Crisis in the emergency department. N Engl J Med 2006;355(13):1300–3. 10.1056/NEJMp068194 17005946

[21] Bhuiya F , Pitts SR , McCaig LF . Emergency department visits for chest pain and abdominal pain: United States, 1999–2008. NCHS data brief, no 43. Hyattsville (MD): National Center for Health Statistics; 2010. 20854746

[22] Venkatesh AK , Geisler BP , Gibson Chambers JJ , Baugh CW , Bohan JS , Schuur JD . Use of observation care in US emergency departments, 2001 to 2008. PLoS ONE 2011;6(9):e24326. Erratum in PLoS One 2012;7(1). 10.1371/journal.pone.0024326 21935398 PMC3173457

[23] Chandra A , Glickman SW , Ou FS , Peacock WF , McCord JK , Cairns CB , An analysis of the Association of Society of Chest Pain Centers Accreditation to American College of Cardiology/American Heart Association non-ST-segment elevation myocardial infarction guideline adherence. Ann Emerg Med 2009;54(1):17–25. 10.1016/j.annemergmed.2009.01.025 19282062

[24] Baigent C , Keech A , Kearney PM , Blackwell L , Buck G , Pollicino C , Efficacy and safety of cholesterol-lowering treatment: prospective meta-analysis of data from 90,056 participants in 14 randomised trials of statins. Lancet 2005;366(9493):1267–78. Erratum in Lancet 2008;371(9630):2084 and Lancet 2005;366(9494):1358. 10.1016/S0140-6736(05)67394-1 16214597

[25] Fraker TD Jr , Fihn SD , Gibbons RJ , Abrams J , Chatterjee K , Daley J , 2007 Chronic angina focused update of the ACC/AHA 2002 guidelines for the management of patients with chronic stable angina. Circulation 2007;116(23):2762–72. Erratum in Circulation 2007;116(23):e558. 10.1161/CIRCULATIONAHA.107.187930 17998462

[26] LaRosa JC , Grundy SM , Waters DD , Shear C , Barter P , Fruchart JC , Intensive lipid lowering with atorvastatin in patients with stable coronary disease. N Engl J Med 2005;352(14):1425–35. 10.1056/NEJMoa050461 15755765

[27] Mosca L , Banka CL , Benjamin EJ , Berra K , Bushnell C , Dolor RJ , Evidence-based guidelines for cardiovascular disease prevention in women: 2007 update. J Am Coll Cardiol 2007;49(11):1230–50. 10.1016/j.jacc.2007.02.020 17367675

